# A co-designed, community-based intensive health behavior intervention promotes participation and engagement in youth with risk factors for type 2 diabetes

**DOI:** 10.3389/fcdhc.2023.1264312

**Published:** 2023-12-01

**Authors:** Julie M. Pike, Kathryn M. Haberlin-Pittz, Basmah S. Alharbi, Susan M. Perkins, Tamara S. Hannon

**Affiliations:** ^1^ Department of Pediatric and Adolescent Comparative Effectiveness Research, Indiana University School of Medicine, Indianapolis, IN, United States; ^2^ Department of Pediatric Endocrinology, Riley Children’s Health, Indiana University Health, Indianapolis, IN, United States; ^3^ Department of Biostatistics and Health Data Science, Indiana University Richard M. Fairbanks School of Public Health, Indianapolis, IN, United States; ^4^ Department of Biostatistics and Health Data Science, Indiana University School of Medicine, Indianapolis, IN, United States

**Keywords:** diabetes prevention, obesity, adolescents, pediatric, lifestyle intervention, physical activity, human-centered design, community-engaged research

## Abstract

**Background:**

Obesity among youth (children and adolescents) is associated with increased risk for youth-onset type 2 diabetes. Lifestyle change can delay or prevent the development of type 2 diabetes, yet real-world implementation of health behavior recommendations is challenging. We previously engaged youth with risk factors for type 2 diabetes, their caregivers, and professionals in a human-centered design study to co-design a lifestyle change program. Here we report the outcomes for this 16-week co-designed lifestyle change program for youth at risk for T2D and their caregivers.

**Research design and methods:**

This single-arm family-based cohort study included youth aged 7-18 years, with BMI ≥85^th^ percentile (overweight or obese) and at least one additional risk factor for type 2 diabetes, and their caregivers. Clinical (BMI, HbA1c), self-reported physical activity, and quality of life outcomes were evaluated at baseline (B), post-intervention (M4), and 1 year (M12) following the intervention.

**Results:**

Seventy-eight youth (mean age 12.4 ± 2.7y, 67% female, 37.8% white) and 65 caregivers were included in the data analysis. Youth baseline BMI z-scores (2.26 ± 0.47) and HbA1c (5.3 ± 0.3) were unchanged at follow up time points [BMI z-scores M4 (2.25 ± 0.52), M12 (2.16 ± 0.58), p-value 0.46], [HbA1c M4 (5.3 ± 0.3), M12 (5.2 ± 0.3), p-value (0.04)]. Youth reported increased physical activity at M4 (p = 0.004), but not at M12. Youth quality of life scores increased at M12 (p=0.01). Families who attended at least one session (n=41) attended an average of 9 out of 16 sessions, and 37 percent of families attended 13 or more sessions.

**Conclusion:**

A co-designed, community-based lifestyle intervention promotes increased physical activity, improved quality of life, maintenance of BMI z-scores and HbA1c, and engagement in youth with risk factors for T2D.

## 1 Introduction

The obesity epidemic is associated with health consequences in children and adolescents, referred to collectively as “youth”. Excess weight gain is strongly linked with the development of insulin resistance, which in combination with pancreatic β-cell dysfunction, plays a key pathophysiologic role in the development of prediabetes and type 2 diabetes (T2D) (1, 2). Risk factors for insulin resistance and T2D include genetics, puberty, and lifestyle factors such as diet high in added sugars and lack of regular physical activity (3). Hazard ratios increase with body mass index (BMI) such that youth with overweight (BMI ≥85^th^ percentile - <95^th^ percentile) are 5.8 times more likely to be diagnosed with T2D, and youth with severe obesity [BMI either ≥120% of the 95^th^ percentile or ≥35 kg/m (2)] are >25 times more likely to be diagnosed with T2D (4). The progression of T2D could be delayed or prevented with intensive health behavior interventions that slow or reverse excessive weight gain, improve nutrition, and increase physical activity (5–8). Moreover, youth with excess body weight and insulin resistance who engage in a minimum of two to three 60-minute sessions of moderate to vigorous activity per week can improve insulin sensitivity and reduce risk for T2D (9).

The efficacy of community-based lifestyle change programs for diabetes prevention in adults is well-established, and research supports lifestyle changes to decrease risk factors for T2D in youth (10–18). Randomized controlled trials (RCT) demonstrate improvements in insulin sensitivity in youth with obesity regardless of weight loss (10, 11, 14). For instance, a RCT of a culturally adapted 3- month intervention, offering 3 times weekly physical activity and nutrition education, significantly improved insulin sensitivity and weight-specific quality of life in youth with obesity (10). Another RCT demonstrated improvements in insulin sensitivity compared to usual clinical care with twice a week physical activity and nutrition classes (11).

While these results are encouraging, efficacious lifestyle interventions for youth with obesity are conducted in the context of research. Effective interventions are culturally tailored, have high participant retention rates, focus on a combination of physical activity and nutrition education, and provide supervised physical activity in controlled settings (19).

Implementation of lifestyle change in real-world settings is a challenge (18, 20, 21). Lifestyle change programs for youth are not supported by healthcare payers and are complicated by socioeconomic factors, community environments, family dynamics, and potentially differing priorities of youth, parents, and clinicians (3, 21–23). Lifestyle change efforts are further challenged and counteracted by insufficient access to healthy foods and safe places for physical activity (24, 25).

There is a lack of evidence regarding effective means to engage youth with obesity and their families in pragmatic, real-world settings (19, 21). Many youth-targeted lifestyle intervention programs are modeled after studies that did not consider unique cultural, societal, environmental, or values of populations of youth at high risk for T2D and their caregivers (3, 19). We previously worked with mothers and their children to modify the National Diabetes Prevention Program curriculum for use with families in a project entitled “ENCOURAGE Healthy Families” (26, 27). From “ENCOURAGE”, we iteratively co-designed “Powerhouse” with partnering families to further address challenges to and preferences for engagement in a health behavior intervention for medically underserved youth and families (28–30). A strategy to develop programs for youth-focused obesity treatment in underserved populations is community-based participatory research using human-centered co-design. Human-centered co-design is defined as a problem-solving method that engages stakeholders in the process of development and the implementation of solutions (31). This process and the findings are previously described (28). Here we report the clinical, physical activity, quality of life, and attendance outcomes for the 16-week (with up to 12 months follow-up) Powerhouse intervention for youth and families.

## 2 Methods

### 2.1 Study design

This is a non-controlled cohort study of a 16-week iteratively co-designed intensive health behavior and lifestyle change intervention for youth, with risk factors for type 2 diabetes, and their caregivers. The study includes four cohorts over approximately two years (January 2017-October 2018). The primary outcomes for the youth in this study are: 1) BMI standard deviation score (BMI z -score); and 2) BMI percentile at each follow-up assessment. The primary outcome for the caregivers is percent change in body weight at each follow-up assessment. Secondary outcomes include: 1) changes in glycosylated hemoglobin (HbA1c) (both youth and caregivers); 2) changes in self-report physical activity (youth only); 3) global health status and quality of life: Pediatric Quality of Life Inventory for youth (via self-report and caregiver proxy) and 36-item Short Form Health Survey (SF-36) for the caregiver; and 4) program attendance rates. This study was approved by the Indiana University Institutional Review Board.

### 2.2 Settings

This research was conducted at three main settings: 1) Indiana University Health Riley Children’s Hospital Youth Diabetes Prevention Clinic (YDPC), an outpatient clinic for youth with obesity and/or prediabetes, 2) a local federally qualified health center serving pediatric patients, and 3) the John Boner Neighborhood Center, in the Near Eastside of Indianapolis where the intervention took place.

### 2.3 Recruitment

Recruitment efforts focused on youth who received medical care at one of the outpatient clinics included above. Research staff promoted the study through healthcare provider meetings, presentations in the community, flyers, and word of mouth. Additionally, the siblings of referred youth were offered enrollment in the study, or the opportunity to attend sessions without participating in the study.

Inclusion criteria were age between 7-18 years, a parent or caregiver willing to participate, BMI in the overweight or obesity categories (BMI ≥85^th^ percentile) and one or more additional risk factors for type 2 diabetes: 1) diagnosis of prediabetes, 2) family history of type 2 diabetes in a first or second-degree relative, 3) history of maternal gestational diabetes, 4) belonging to a minoritized race/ethnic group, 5) conditions associated with insulin resistance as assessed by a physician (acanthosis nigricans, hypertension, dyslipidemia, polycystic ovarian syndrome, small for gestational age birth weight), and 6) English speaking. Exclusion criteria were inability to participate in in-person group meetings or having a diagnosis of youth-onset diabetes or any other chronic medical condition that would preclude participation in group meetings.

At least one caregiver was required to participate in the program with the youth participant. Youth were screened for eligibility during the recruitment process and again at baseline data collection. Siblings who did not meet the eligibility criteria could participate in the program but were excluded from data analysis. All the adult participants (≥18 years old) signed an informed consent and youth participants signed an informed assent prior to study participation.

### 2.4 Intervention

Powerhouse is a 16-week intensive health behavior and lifestyle change intervention to decrease modifiable risk factors for type 2 diabetes in youth and their caregiver. Powerhouse was co-designed using a participatory, human-centered co-design approach as previously described (28). Briefly, we previously partnered with diabetes care professionals, community stakeholders, parents, and youth with obesity and risk factors for T2D in a series of sessions designed to 1) better understand environmental factors and program characteristics that could promote participation; 2) learn how best to include physical activity and nutrition education to meet the needs of participants; and 3) co-design the learning activities. We found that participants wanted a program that felt affirming, positive, and fun (28). They wanted interactive learning activities that incorporated a sense of play and trying new things (28). Participants appreciated rewards, and they wanted opportunities to build relationships with other families in the group (28). The co-design study was a separate study from the Powerhouse intervention.

Community partners provided access to meeting space, exercise amenities, cooking facilities, and an urban garden.

#### 2.4.1 Group sessions

Powerhouse met weekly for 16 weeks. Each session lasted two hours. Youth and adult participants engaged in physical activity for the first 45-60 minutes of the session. Youth participated in supervised, age-appropriate game-based play (i.e., capture the flag, soccer, tag). Parents participated in conventional exercise sessions (elliptical machine, stationary bike, walking, strength equipment, stretching/yoga). The second hour included a nutrition activity, goal setting, and a shared meal that was either donated by a community partner or prepared together during the group session. Participants received periodic prize drawings for setting and tracking progress with weekly goals and completing food and activity logs. A registered dietitian and a certified health education specialist facilitated the sessions and were supported by undergraduate and graduate-level students.

#### 2.4.2 Curriculum

Nutrition and physical activity recommendations were adapted from the Expert Committee Recommendations Regarding the Prevention, Assessment and Treatment of Child and Adolescent Overweight and Obesity (32) and the National Diabetes Prevention Program (https://www.cdc.gov/diabetes/prevention/index.html). The Expert Committee recommendations include avoiding sugar-sweetened beverages, encouraging fruits and vegetables, eating breakfast daily, limiting restaurant and fast-food meals, using portion control, encouraging family mealtimes, engaging in 60 minutes of daily physical activity, and limiting screen time (32). The National Diabetes Prevention Program is a proven lifestyle change intervention for decreasing T2D risk that has been successfully adapted for a variety of populations and delivery modes (15, 27). The ENCOURAGE Healthy Families program, a DPP adaptation for high-risk mothers and their children, was the foundation for the co-design of a diabetes prevention curriculum for youth and their caregivers (26, 27).

Recommendations for food choices, physical activity, and goal setting remained constant for all cohorts. However, the implementation of learning activities followed the principles of iterative design, a process of continuous prototyping, testing, and adjusting to meet the needs of participants. For example, participants in the first cohort valued cooking sessions over other group-based interactions. Therefore, facilitators incorporated more cooking sessions in cohorts two through four. Participants indicated a lack of access and knowledge about fresh produce, so Powerhouse offered gardening activities and free produce for cohorts three and four. Powerhouse also added gentle yoga for adults in cohorts 3 and 4 based on the preference for low-intensity physical activity and stress-reducing mindfulness activities.

### 2.5 Data collection

Research staff collected clinical measurements and surveys at baseline (B), 16-weeks (M4), and 12 months (M12).

#### 2.5.1 Clinical measurements

Height and weight measurements were completed in private rooms (caregiver and youth of the same family together) to ensure privacy and confidentiality. Height was measured to the nearest 0.1 cm using a stadiometer (SECA Model 213 1821009). Weight was measured to the nearest 0.1 kg with a digital scale (Healthometer Professional Model 349KLXN, Dectecto Scale Model 758C).

Research staff calculated BMI using the National Institutes of Health online BMI calculator (https://www.nhlbi.nih.gov/health/educational/lose_wt/BMI/bmi-m.htm) or the Centers for Disease Control online BMI percentile calculator for children and adolescents (https://www.cdc.gov/healthyweight/bmi/calculator.html). Research staff calculated the percentage of the 95^th^ percentile to better quantify measures of BMI about the 95th percentile: [(participant’s BMI – BMI at 95^th^ percentile)/(BMI at 95^th^ percentile)] x100 (https://peditools.org/growthpedi/index.php).

HbA1c was measured using point-of-care testing (Alere Afinion AS100 machine, Alere, Orlando, FL) by three trained members of the research staff and according to product specifications.

#### 2.5.2 Physical activity and quality of life surveys

Participants reported physical activity and quality of life on paper surveys, and research staff entered data into the REDCap electronic data collection system (33).

##### 2.5.2.1 Fels Physical Activity Questionnaire for Children

The Fels Physical Activity Questionnaire for Children (FELS PAQ) measures physical activity in specific domains (sports, leisure, and chores) for youth 7-18 years of age (34). Total physical activity scores are the sum of sports, leisure activities, and chore indexes. Higher total Fels PAQ scores indicate greater levels of physical activity.

##### 2.5.2.2 Pediatric Quality of Life Inventory

Pediatric Quality of Life Inventory (PedsQL 8-12, PedsQL 13-18) is a 23-item tool that measures general health related quality of life in physical and psychosocial domains (35). Higher scores indicate greater quality of life.

##### 2.5.2.3 36-item Short Form Health Survey

The 36-item Short Form Health Survey (SF 36) consists of eight domains (physical functioning, role physical, bodily pain, general health, vitality, social functioning, role emotional, mental health) that assess the health-related quality of life of adults (36). Higher scores indicate better health status.

#### 2.5.3 Attendance

Research staff recorded weekly session attendance in a Microsoft Excel (2018) spreadsheet. Attendance is reported as family attendance. This is defined as the attendance of one or more family members.

### 2.6 Compensation

Both youth and caregivers in the first two cohorts received a $20, $30, and $40 gift card at baseline, M4, and M12, respectively. After receiving additional funding for the project and based on feedback from participants in cohorts 1 and 2, monetary incentives increased for cohorts 3 and 4. Cohorts three and four received a $30, $40, $50 gift card at baseline, M4, and M12, respectively.

### 2.7 Data analysis

All data analysis was conducted using SAS version 9.4 (SAS Institute, Inc., Cary, NC). Linear mixed-effect models estimated changes in the two primary outcomes for the youth in this study: 1) BMI z-score; and 2) BMI percentile at each follow-up assessment. If the overall test for time was significant in the linear mixed models, pairwise *t*-tests were conducted within the linear mixed model to see which time points differed. For percent change in weight for the caregiver, 4- and 12-month percent changes were tested for equality to zero using one-sample *t*-tests. Secondary outcomes measured with linear mixed-effect models were: 1) changes in HbA1c (both youth and caregivers); 2) changes in self-report physical activity (youth only); and 3) global health status and quality of life: Pediatric Quality of Life Inventory for youth (via self-report and caregiver proxy) and SF-36 for the caregiver. All linear mixed effect models included a fixed effect for time (treated as a categorical variable with baseline as a reference) and a participant-nested-within-family random effect (with unstructured correlation) to allow for both the repeated measures over time and the correlation between multiple participants within a family. Separate models were fit for youth and caregivers. For questionnaire subscales, we also adjusted the overall p-value for multiple testing using the Bonferroni approach.

To assess for bias due to dropout, two-sample *t-*tests were used to compare participants who completed baseline and both follow-up time points with those who completed baseline and only one follow-up timepoint. Attendance was reported with descriptive statistics.

## 3 Results

### 3.1 Referrals

The study received 320 referrals from primary care providers (50.3%), the Youth Diabetes Prevention Clinic (33.1%), self-referral (8.8%), and sibling-referral (7.8%). Sibling-referral refers to youth who participated in the program because a sibling was a participant. Reasons for not enrolling in the program included being unable to reach, declined participation after learning about the study, non-English speaking, and not able to attend the scheduled baseline data collection date.

Eighty-five youth and 67 caregivers consented to participate. Two youth (and their caregivers) were not included in data analysis due to missing BMI data. Five participating siblings did not meet the baseline BMI criteria, participated in sessions, but were excluded from data analysis.

### 3.2 Participants

Youth (n=78) were ages 7–18 years old (12.4 ± 2.7 years), 67% female. Forty-eight families had 1 caregiver enrolled, 7 families had two caregivers enrolled, and one family had three caregivers enrolled. Forty families had 1 child enrolled, 11 had two, 4 had three, and one had four. Of the 78 youth, 43 were between 8 and 12 years old and 34 were between 13 and 18 years old, and one child was less than 8 years old. The cohorts were predominantly of minority race/ethnicity: 37.8% were white, 35.6% were black, 4.9% were Latino, and 21.7% were other races. There were no demographic differences for participants who completed 12-month follow-up or only 6-month follow-up.

### 3.3 Clinical outcomes

In linear mixed models, all clinical outcomes were normally distributed except for BMI percentile scores for youth. Ninety-six percent of BMI percentile values were above the 90^th^ percentile; 57.5% of the BMI percentile values were at the 99^th^ percentile and 3.9% were above the 99^th^ percentile. For youth with BMI measures at two times points, BMI percentile scores decreased for 13 youth, did not change for 36 youth, and increased for 15 youth.

BMI and HbA1c outcomes are shown in **Table 1**. In youth, there were no changes observed for BMI z-score (p = 1.00) from baseline to M4 or M12. *Post hoc t*-tests for HbA1c indicated the M4 mean did not differ from baseline (p = 0.72), the M12 mean was lower than baseline (p-value = 0.04), and the M12 mean was also lower than the M4 mean (p-value = 0.02). For caregivers, body weight, percent change in body weight, and HbA1c did not change.

**Table 1:** BMI and HbA1c in youth and caregivers.

	Baseline	Month 4	Month 12	P-value
Youth				
BMI z-score	2.26 ± 0.47 N=78	2.25 ± 0.52 N=57	2.16 ± 0.58 N=44	0.46
HbA1c (%)	5.3 ± 0.3 N=76	5.3 ± 0.3 N=57	5.2 ± 0.3 N=45	0.04*
Caregivers				
BMI (kg/m^2^)	37.5 ± 8.9 N=64	38.1 ± 8.7 N=48	36.9 ± 8.7 N=35	0.70
Weight (kg)	102.3 ± 23.4 N=64	103.4 ± 22.3 N=48	101.1 ± 21.8 N=35	0.80
Weight change (%)		0.66 ± 7.07 N=47	0.72 ± 3.19 N=34	0.52
HbA1c (%)	5.6 ± 1.1 N=62	5.7 ± 1.2 N=46	5.8 ± 1.6 N=35	0.80

Data are means ± SD.

*B, M4 p=0.72; B, M12 p=0.04; M4, M12 p=0.02.

BMI z-score: BMI standard deviation score.

HbA1c (%): glycosylated hemoglobin.

BMI (kg/m^2^): body mass index.

### 3.4 Physical activity and quality of life

In the linear mixed models, all physical activity and quality-of-life outcomes were normally distributed. **Table 2** displays the means and standard deviations for the FELS PAQ and PedsQL questionnaires and subscales. The FELS PAQ total score, work, and leisure indices, did not change from baseline to M4 or M12. The FELS PAQ sport index score indicated an overall time effect (adjusted p = 0.02). *Post hoc t*-tests indicated the M4 mean was greater than baseline (p = 0.004), and the M12 mean was no different from baseline and lower than the M4 mean (p = 0.01).

**Table 2:** Physical activity and quality of life scores in youth.

	Baseline	Month 4	Month 12	P-value
	N=78	N=57	N=45	
FELS PAQ total	2.78 ± 0.65	2.79 ± 0.87	2.79 ± 0.53	0.08
Sports Index	2.73 ± 0.87	3.04 ± 0.89	2.74 ± 0.91	0.02^a^
Leisure Index	2.03 ± 0.83	2.06 ± 0.82	2.19 ± 0.84	0.46
Work Index	3.62 ± 1.07	3.79 ± 0.87	3.43 ± 1.01	0.56
PedsQL, youth respondents				
Total Score	68.4 ± 17.1	71.8 ± 13.7	73.4 ± 14.3	0.009^b^
Physical Summary	75.6 ± 16.8	77.2 ± 16.0	80.4 ± 12.9	0.04^c^
Psychosocial Summary	62.4 ± 18.9	68.9 ± 16.3	69.7 ± 16.9	0.02^d^
Emotional subscale	61.0 ± 23.1	65.0 ± 21.3	64.5 ± 23.6	0.20
Social subscale	67.2 ± 26.3	72.9 ± 22.0	75.0 ± 17.0	0.04
School subscale	65.4 ± 20.8	68.8 ± 18.4	68.7 ± 20.0	0.35
PedsQL, caregiver respondents (N=76)				
Total Score	65.7 ± 16.5	66.8 ± 13.9	70.1 ± 15.1	0.17
Physical Summary	68.5 ± 20.7	69.5 ± 20.8	73.7 ± 20.0	0.41
Psychosocial Summary	64.4 ± 16.9	65.3 ± 13.5	68.1 ± 15.4	0.13
Emotional subscale	61.4 ± 22.0	63.1 ± 21.0	64.9 ± 19.2	0.41
Social subscale	67.9 ± 24.5	67.2 ± 19.3	72.3 ± 20.5	0.55
School subscale	63.4 ± 16.5	65.5 ± 18.2	67.5 ± 21.2	0.66

a B, M4 p<0.01; B, M12 p=0.93; M4, M12 p=0.01.

b B, M4 p=0.13; B, M12 p=0.002; M4, M12 p=0.10.

c B, M4 p=0.74; B, M12 p=0.02; M4, M12 p=0.04.

d B, M4 p=0.03; B, M12 p<0.01; M4, M12 p=0.48.

FELS PAQ: Fels Physical Activity Questionnaire for Children.

PedsQL, youth respondents: Pediatric Quality of Life Inventory for youth, self-report.

PedsQL, caregiver respondents: Pediatric Quality of Life Inventory for youth, caregiver proxy.

PedsQL measures from youth participants showed improvements in total, physical, and psychosocial scores. The PedsQL total scale score increased from baseline to M12 (p = 0.01). The M4 mean was not different than baseline (p = 0.13), but the M12 mean was greater than both the baseline (p = 0.002) and M4 means (p = 0.01). The PedsQL physical summary score increased from baseline to M12 (p = 0.04). The M4 mean was not different than baseline, but the M12 mean was greater than both the baseline (p = 0.02) and M4 means (p = 0.04). The psychosocial summary score also improved (p = 0.02) with increases from baseline to M4 (p = 0.03) and baseline to M12 (p = 0.01). There were no significant changes for the PedsQL emotional, social, or school subscales after adjusting the p-values for multiple testing. For the PedsQL parent proxy questionnaire, measures did not indicate any change in caregivers’ impressions of youth quality of life, though there was a pattern of increasing scores like that seen in the youth-response questionnaires. The Short-Form Health Survey (SF-36) administered to caregivers did not indicate change in quality of life, or subscales of quality of life (data not shown).

### 3.5 Intervention attendance

**Figure 1:**
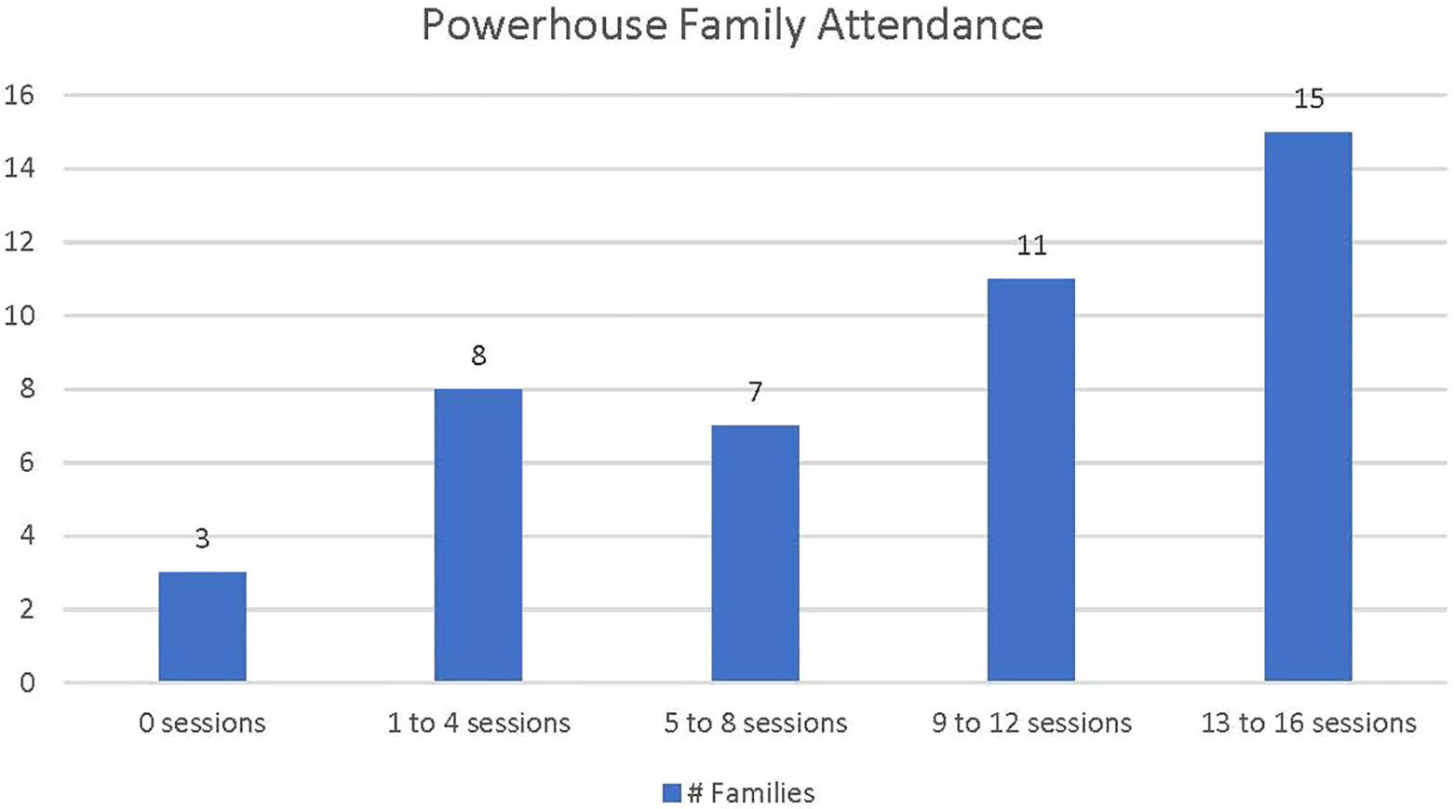
Powerhouse family attendance. Family attendance is defined as family attendance of one or more family members.

Attendance data is based on forty-four families who attended at least one follow up data collection session, either M4 or M12 (**Figure 1**). Three families did not attend any Powerhouse sessions. Families who attended at least one session (N=41), attended an average of nine sessions (18 contact hours). Nineteen families completed eight or fewer sessions. Twenty-six families completed more than half of the sessions, and fifteen of those families completed 13 or more sessions (26 or more contact hours).

## 4 Discussion

The present study evaluated the engagement and health-related outcomes of Powerhouse, as conducted in a pragmatic, family-based fashion in a community-setting. This was an important next step to increase access to an intensive health behavior intervention that could be delivered outside of the typical clinical care setting. We demonstrated that a community-based approach could achieve increased rates of participation in physical activity and improvements in quality of life, while maintaining BMI z-score and favorable HbA1c among youth who participated in Powerhouse.

Our findings of maintained BMI z-scores and maintained or decreased BMI percentile measures from pre- to post-intervention (12 months) are consistent with other lifestyle interventions for youth with obesity. Clinically meaningful weight loss based on observational and interventional studies has been defined by some as a BMI z-score reduction between 0.20–0.25 standard deviations over 6–12 months (37, 38). The lack of increase in BMI z-scores could perhaps be a positive outcome given the natural history of upward trajectory of BMI in youth-onset obesity into adulthood (39–41). A meta-analysis found that lifestyle interventions for youth obesity were not generally associated with a change in BMI outcomes (19). Nevertheless, there is a wide range of metabolic and cardiovascular benefits associated with participation in lifestyle-based intervention regardless of change in BMI (19). Savoye et al. (11, 42, 43) have shown in multiple studies that a family-based, lifestyle intervention tailored for inner-city minority children and their families (Bright Bodies) results in clinically significant improvements in insulin sensitivity, blood glucose, and cholesterol values, while BMI or BMI z-scores are maintained close to baseline or reduced by less than the 0.2 standard deviation change reported to be clinically significant. While the Bright Bodies intervention has been expected to reduce a participants BMI by a very modest 1.67 kg/m^2^ per year, the impact of the intensive lifestyle intervention on reducing obesity-related healthcare expenditures associated insulin resistance is projected to be $1126 per person over 10-years compared with the clinical control intervention (44, 45).

The mean baseline HbA1c for youth in Powerhouse was within normal values which limited our ability to detect a positive change. Maintenance of HbA1c in this population is favorable given that most youth were of age to be in puberty, and all had additional risk factors for T2D (i.e., obesity, acanthosis nigricans, family history). There is substantial evidence that pancreatic β-cell function worsens over time in youth with impaired glucose tolerance or recently diagnosed T2D despite efforts to preserve β-cell function with diabetes treatments (1). Thus, interventions are likely needed prior to the development of dysglycemia in youth at risk for T2D. The resolution of physiological pubertal insulin resistance may have contributed to optimal HbA1c levels for some older youth.

In Powerhouse, we focused on increasing physical activity and adopting healthier food choices rather than caloric restriction for weight loss. Indeed, we documented and observed that youth participated in moderate to vigorous physical activity during weekly sessions, and youth self-reported greater sports-related physical activity at M4 and improved physical and psychosocial quality of life at M12 and at M4 and M12, respectively. Increased sports-related physical activity at M4 is likely reflective of participation in Powerhouse as the FELS PAQ sports index includes the types of games-based play offered at Powerhouse sessions, and sports-related physical activity returned to baseline levels after Powerhouse participation was completed (M12). There is evidence that increasing levels of physical activity are associated with positive longer-term health outcomes, regardless of BMI. Lee et al. (46) reported positive associations between resistance exercise, aerobic exercise, or both, and insulin sensitivity while BMI remained relatively constant. For both youth and adults, regardless of BMI, higher levels of physical activity and lower levels of sedentariness are associated with better insulin sensitivity, clinically significant reduction in cardiometabolic risk profiles, and superior long-term health physical and mental health outcomes (46–50).

Because our work was pragmatic, we only evaluated frequency, intensity, and type of physical activity via self-report questionnaire. Although once weekly hourly bouts of physical activity do not meet the national guidelines for recommended amounts of physical activity, we demonstrated the ability to facilitate moderate to vigorous physical activity under direct observation in a cohort of youth who were getting little to no physical activity at baseline.

A meta-analysis revealed, on average, youth spend just 27.8 min (4.4 min/hour) engaging in moderate to vigorous activity at school (51). Youth in afterschool programs get a bit more moderate to vigorous activity (11.7 min/hour), but still do not meet the recommended 60 min per day (51). This underlines the need for ongoing, free structured community- and school-based opportunities for all levels of play-based, group physical activity.

Powerhouse had strong participant engagement, which might be associated with the reported improvements in quality of life. Improvements in quality of life are consistent with findings from other lifestyle change programs (14, 52). Participation in a Diabetes Prevention Program for Latino youth improved weight related quality of life scores, despite a lack of weight loss (52). This is significant as obese youth report significantly impaired quality of life compared to their lean counterparts (53). Families who participated in Powerhouse (n=41) attended an average of 9 sessions (18 contact hours) and 37% of those families attended 13 or more sessions (≥26 contact hours). A real-world adaptation of a family-based weight management program reported improvements in BMI with a mean dose of 12 contact hours (45). Reports of adherence to pediatric community-based programs are sparse. Participant session attendance is also not consistently reported in research studies, and program attrition varies widely. One lifestyle intervention for youth living with T2D reported a 60% session completion for participants, whereas, a diabetes prevention program for Latino youth reported 82.5% retention at 12 months (10, 54). Most studies of pediatric weight management programs report only 20% retention (55). A variety of complex factors contribute to program attrition including unmet participant expectations and needs, which we attempted to address in the codesign process for the intervention (29, 30, 56). This approach may be especially important for underserved youth and their families (19, 20).

Many family-focused diabetes prevention programs reported in the literature include caregivers in the lifestyle change sessions, but do not measure their health-related outcomes, making caregiver outcomes a meaningful aspect of this study. It is apparent in the pediatric weight management literature that parents serve as change agents in the home (57). Lack of change for caregivers’ measures may be related to the fact that most participants were recruited through physician referrals for youth. Caregivers may have viewed lifestyle change as a priority for youth rather than themselves, especially as most caregivers had HbA1c values within normal limits. Absence of caregiver BMI change likely influenced youth BMI as parental weight loss in family-based obesity treatment programs predicts weight change in youth (58).

### 4.1 Limitations

This study had limitations. Medical evaluation of other comorbidities, performing oral glucose tolerance testing or tests of insulin sensitivity was outside the scope of this pragmatic, community-based intervention. This study also had an overall attrition for both youth and caregivers at M12 of 45%, which limits are ability to interpret outcomes for the cohort.

## 5 Conclusion

An iteratively co-designed, community-based lifestyle intervention successfully engaged families and was associated with increased levels of physical activity, improved quality of life, and maintenance of BMI z-scores and HbA1c in youth with risk factors for T2D.

## Data availability statement

The raw data supporting the conclusions of this article will be made available by the authors, without undue reservation.

## Ethics statement

The studies involving humans were approved by Indiana University Institutional Review Board. The studies were conducted in accordance with the local legislation and institutional requirements. Written informed consent for participation in this study was provided by the participants’ legal guardians/next of kin.

## Author contributions

JP: data curation, investigation, methodology, project administration, writing – original draft. KH-P: data curation, investigation, methodology, project administration, writing – review & editing. BA: formal analysis, data curation, writing – review & editing. SP: writing – review & editing. TH: conceptualization, funding acquisition, investigation, methodology, resources, supervision, writing – review & editing.
